# The Tridirectional Relationship among Physical Activity, Stress, and Academic Performance in University Students: A Systematic Review and Meta-Analysis

**DOI:** 10.3390/ijerph18020739

**Published:** 2021-01-16

**Authors:** Kathrin Wunsch, Janis Fiedler, Philip Bachert, Alexander Woll

**Affiliations:** Institute of Sports and Sports Science, Karlsruhe Institute of Technology, 76131 Karlsruhe, Germany; Janis.Fiedler@kit.edu (J.F.); Philip.Bachert@kit.edu (P.B.); Alexander.Woll@kit.edu (A.W.)

**Keywords:** academic stress, exams, exercise, student’s health, grade point average

## Abstract

Higher education students often suffer from physiological and psychological health problems caused by stress, which may negatively impact their academic performance (AP). Physical activity (PA) can be a promising strategy to buffer these stress-induced complaints. Therefore, the aim of this investigation was to summarize evidence for the tridimensional construct of PA, stress, and AP, as well as to quantify the relationships among these variables. Five databases (PubMed, Scopus, SMEI, ERIC, and Web of Science) were systematically searched in November 2019 for publications that examined PA, stress, and AP of university students, without any restrictions regarding the publication period. The systematic review includes four original research studies with a moderate-to-high risk of bias. Results of included studies were narratively summarized and quantified in a meta-analysis using random effect models. Whereas study results point to a positive relation between PA and AP, relationships between PA and stress seem to be negative, while the relation between stress and AP is undecided. The meta-analysis found no significant associations and considerable heterogeneity of the results. Findings indicate a research gap concerning the connection of PA, stress, and AP in university students. Future studies should use validated measuring tools and consider the timepoint of data collection in order to extract truly stressful periods.

## 1. Introduction

In recent years, a growing body of research has emerged, showing that a major concern of higher education students is suffering from physiological and psychological health problems. Stewart-Brown and colleagues showed that one-third of university students reported at least one long-standing illness [1]. More recent investigations revealed a similar amount of students suffering from mental issues, showing that student life can be a cause of distress, as students report higher distress levels than their non-student peers [2], and high levels of stress impact the quality of life [3]. An obvious causal factor being accountable for (periodic) high levels of student stress is the examination period at the end of each semester, thus forming a real-life stress situation. Particularly, this phase causes immediate negative effects on health-related outcomes, such as poor sleep quality and well-being [4], which are positively related to cognition and academic achievement or academic performance (AP) [5,6]. Hence, AP is commonly affected in high-stress periods, where the highest cognitive functioning is required.

Physical activity (PA) and exercise are known to be stress-buffering behaviors, as engagement in regular PA can buffer negative effects of stress on health, which is postulated by the stress-buffering hypothesis [7,8]. In general, PA is known to have several positive effects on physiological and psychological stress-related parameters. There is early evidence to support the stress-modulatory effect of PA. Brown and Siegel [9] revealed that sedentary participants with high stress levels had an elevated disease incidence and that physically active participants with high stress were protected against the stress-induced increases in disease incidence. Furthermore, stress level, anxiety, and depression of university students increases as their sedentary time increased [10]. Similar results have been drawn regarding psychological health [8]. Klaperski, Seelig and Fuchs showed that PA exhibits a health-protective effect, especially under chronic stress conditions [11]. Overall, Nguyen-Michel, Unger, Hamilton, and Spruijt-Metz revealed a significant negative relationship between PA and stress in a student sample [12]. Especially in high-stress periods like examination phases, PA decreases and may therefore not provide a suitable stress-buffer for students.

Hence, it is especially the examination phase in which students could profit from good stress-buffering abilities, as high amounts of perceived stress are known to diminish cognitive functioning in students [13], which in turn is highly correlated to AP [14]. Studies revealed significant negative correlations between perceived stress and AP in students, especially within examination periods at the end of a semester [15,16,17].

Literature including child and adolescent populations suggests that PA positively influences AP, as more active students show better AP [18,19], even if there is only limited evidence [20,21]. Interestingly, this association is commonly examined in pupils (school children) and needs to be further addressed in university student populations, particularly because the majority of university students do not meet the recommendations for PA [22,23,24].

Until today, there has been a lack of knowledge on the relationship between PA, stress, and AP in university students. However, especially this tridirectional relationship is of interest, as stress burden is exceptionally high in academic examination phases, where cognitive function demands are concomitantly high to achieve best AP [13,14]. As the cross-stressor-adaptation hypothesis [25] posits that regular PA (as a stressor itself) elicits unspecific adaptations enabling humans to also show lower reactions to heterotypic stressors (i.e., psychosocial or cognitive stressors, like examinations periods; [26]), it is of high interest to also include AP as an outcome variable into this consideration. Based on knowledge of the bidirectional relationships of PA, stress and AP, it can be hypothesized that PA serves as a mediator or moderator in the relationship between stress and AP. To gain insights into the possible stress-buffering and cross-stressor-adaptation effects of PA and simultaneous benefits to AP in stressful periods in university students, the current investigation aims to form a systematic review and meta-analysis to expand upon research on bidirectional relationships of PA and stress, stress and AP, as well as PA and AP, while focusing only on studies assessing all three variables in order to gain insights into the tridirectional relationship. This is especially important in terms of public health, as policymakers and universities may profit from results in order to account for student-specific, PA-based health interventions to increase AP in real-life stress situations.

## 2. Methods

This systematic review was performed and reported following the Preferred Reporting Items for Systematic Reviews and Meta-Analysis (PRISMA) guidelines [27].

### 2.1. Eligibility Criteria

Primary source and peer-reviewed articles published in English were eligible for inclusion in this systematic review and meta-analysis if data were presented for PA, stress, and AP simultaneously. Specific eligibility criteria included the following: types of participants: university students. Types of outcome measures: each dependent variable had to be measured and reported, i.e., PA (via self-report or accelerometry/pedometers), stress (via self-report or any physiological measure), and AP (via self-report or grades). Study design: no restrictions. Exclusion criteria: articles were excluded if they did not meet inclusion criteria or did not include findings related to inclusion criteria (i.e., measured PA, but failed to compare with stress or AP).

### 2.2. Information Sources

Five different databases were used for literature search: PubMed, Scopus, SMEI, ERIC, and Web of Science. Search terms were applied to meet the specific demands of each database. Two authors performed the search independently. In case of divergence or ambiguity, results were discussed until a consensus was reached.

### 2.3. Search

Search terms were defined through group discussion among the research team and were used in each database without any restriction regarding the publication period to identify potential articles with abstracts for review in November 2019. Using the PICO search tool [28], the following key search-terms were identified: university students, PA, academic stress and AP. In combination with synonyms of all components, combined searches were performed in the different databases. The specific search terms for each database can be found in Supplementary Table S1. Identified publications were then transferred to Citavi (version 6.5.0.0) for further processing.

### 2.4. Study Selection

Title and abstracts of retrieved studies were independently assessed for eligibility for inclusion in the review by two authors. Disagreements regarding eligibility for inclusion were resolved via consensus among all authors. Full-text articles for eligible abstracts were retrieved and reviewed by the same two authors prior to inclusion in the review. A Microsoft Excel spreadsheet was developed to track the eligibility status.

### 2.5. Data Collection Process and Data Items

Extracted data were entered into an Excel spreadsheet. Relevant data were extracted from each manuscript by one author and the coding was verified by a second author. Disagreements were resolved by discussion among these authors. Data extracted from each article included general information (authors, year, country), basic information on methods (aim, study design, sample characteristics, sampling time, methods used regarding PA, stress and AP) as well as results (direct association statistics, central results, and sub findings). If different measurement methods (e.g., self-reported success vs. grade point average (GPA) or self-reported vs. device-measured PA) were used in the studies, the ones which were most comparable between the studies (i.e., self-reported PA and GPA) were included. For each relationship, relevant effect sizes were retrieved.

### 2.6. Risk of Bias in Individual Studies and Risk of Bias across Studies

To assess the risk of bias across studies, funnel plots were compiled using R [29]. For the assessment of the risk of bias in individual studies, the Appraisal Tool for Cross-Sectional Studies (AXIS) was used [30]. To quantify the risk of bias of individual studies, a scoring method has been adapted [31]. Following this method, the studies were categorized as very low risk of bias if they scored correctly on at least 19 out of 20 of the questions, low risk of bias if they scored 17 or 18 out of 20; moderate risk of bias if they scored 15 or 16 out of 20 and high risk of bias if the studies scored 14 or less.

### 2.7. Summary Measures

In order to perform the meta-analysis, all effect sizes were extracted from the original studies and transformed into correlation coefficients. If betas or effect size estimates were reported in the studies and the original correlation coefficients could not be obtained, the betas and effect size estimates were treated as correlation coefficients [32]. F-values from ANOVAs were transformed to correlation coefficients using the online platform psychometrica [33]. X^2^ values were transformed to Cramer’s V using the following formula:

$$\sqrt{\frac{X²}{n\left(K-1\right)}}$$

Here, K is the number of rows or number of columns, whichever shows the smaller number [34]. Cramer’s V was treated as a correlation coefficient subsequently [35].

### 2.8. Additional Analyses and Synthesis of Results

An original analysis of the tridirectional relationship was not possible due to missing information (see results of individual studies). Contacting the authors to provide the missing information was not successful. Therefore, articles were grouped by the respective dependent variable. Hence, three datasets were derived, based on correlational findings on the relationships of: (1) PA and AP, (2) PA and stress, and (3) AP and stress.

To gain a basis for meta-analytical interpretation, all effect sizes were transformed into correlation coefficients (see summary measures). These were Fishers-z-transformed to gain comparable results. A random-effects model was used for the three multilevel meta-analyses concerning the relationship of (1), (2), (3) (see above). The results were interpreted following [36]. Based on empirically derived effect size distribution, correlation coefficient values of 0.12, 0.24, and 0.41 should be interpreted as small, medium, and large effects for social psychology studies.

The Q-test for heterogeneity [37] is reported to display the amount of heterogeneity among with the I^2^ value [38], where values of 0% to 40% indicate no important, 30% to 60% moderate, 50% to 90% substantial, and 75% to 100% considerable heterogeneity [39]. The analysis was carried out using R (version 3.6.1) [29] and the metafor package (version 2.1.0) [40].

## 3. Results

### 3.1. Study Selection

**Figure 1:**
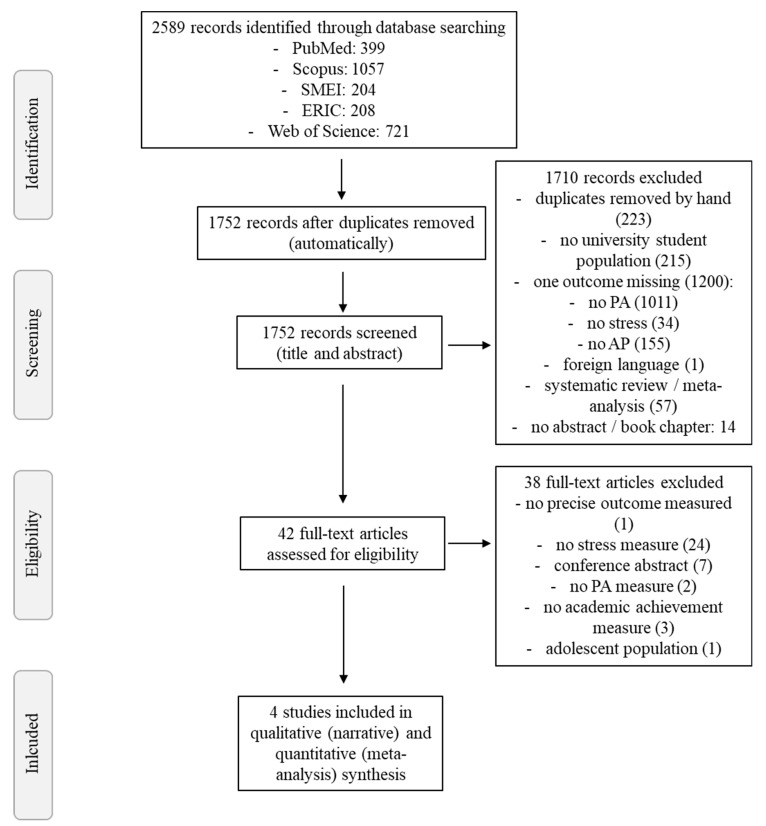
Flow chart of study selection process. Note: AP: academic performance; PA: physical activity.

Out of the 2589 studies initially located and downloaded, 837 doublets were automatically removed in Citavi. Based on title and abstract screening, an additional 1710 studies were excluded, which resulted in 42 studies for full-text screening. In this step, 38 studies were excluded due to not meeting inclusion criteria. Thus, a total of four original research studies were included in this meta-analysis [41,42,43,44]. Please see Figure 1 for the full study selection process and reasons for exclusion during screening.

### 3.2. Study Characteristics

Two out of the four studies were conducted in the USA, one in France, and one in China. The studies included three cross-sectional and one cohort study and were published between 2011 and 2018. Participants were undergraduate students and sample sizes ranged from 203 [41] to 1071 [44], resulting in a total sample size of 1952 participants (*n*_female_ = 1220, *n*_male_ = 732) throughout included studies. Detailed study characteristics can be retrieved from Table 1.

### 3.3. Risk of Bias within Studies

While quantifying the risk of bias by the AXIS tool, one study was rated at moderate risk of bias (15/20) [43] and three studies at high risk of bias (14/20) [41,42,44]. The main weaknesses were the lack of sample size justification, not addressing non-responders, not clarifying funding sources or conflict of interest, and not describing the ethical approval or consent of participants. For more information on risk of bias assessment see Table 2.

### 3.4. Risk of Bias across Studies

**Figure 2:**
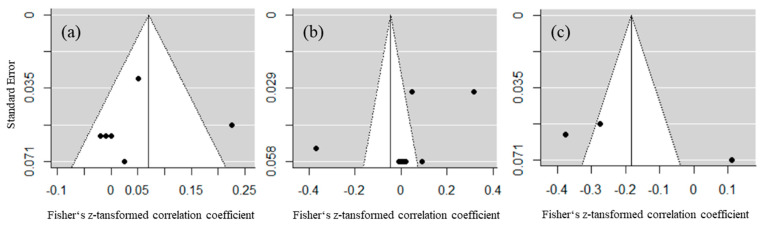
Funnel plots for publication bias between studies for the bidirectional relationships. (**a**) Physical activity and academic performance; (**b**) physical activity and stress; (**c**) stress and academic performance.

Publication bias across studies was assessed using funnel plots for the three bidirectional relationships. Statistical tests of publication bias were not conducted due to the small number of studies [28]. Visual inspection of funnel plots (Figure 2) indicated a small publication bias for the relationship of PA and AP, but high publication bias for the relationships of PA and stress as well as for stress and AP as of the visible asymmetry of effect sizes.

### 3.5. Study Characteristics

Included studies used heterogeneous designs. Whereas Decamps and colleagues [44] and Kayani and colleagues [43] used cross-sectional designs, Rettinger and colleagues [42] gained longitudinal data, but performed their analyses from averaged data across measurement points in a cross-sectional manner, therefore losing information from the repeated-measures design. Ruthig and colleagues [41] were the only to use a longitudinal design and analyses and therefore the only ones to account for time-based alterations.

Studies also differed in assessment methods, especially regarding PA measures. Whereas Kayani and colleagues [43] and Rettinger and colleagues [42] used the short form of the International Physical Activity Questionnaire [45] consisting of seven items measuring PA, Decamps and colleagues [44] and Ruthig and colleagues [41] both used a single-item to assess PA. However, they did not ask for PA in general, but for sports or exercise activity, therefore excluding PA like active transport or gardening, which are not classified as sports or exercise but also important in relation to PA. Analogous, measures for stress also differed between the studies. However, only validated and multi-item questionnaires were used by all studies [46,47,48,49]. Regarding AP, Kayani and colleagues [43] and Rettinger and colleagues [42] both used GPA measures in form of average course grades over the past semester, providing a general view of AP. Decamps and colleagues [44] assessed AP with a dichotomous (only nominal scaled) variable (i.e., fail vs. success), and Ruthig and colleagues [41] used a single course grade for operationalization, therefore not portraying the general AP of students.

While Decamps and colleagues [44], Kayani and colleagues [43] and Rettinger and colleagues [42] aimed to recruit a representative student sample, Ruthig and colleagues [41] investigated psychology students, resulting in a homogenous sample and limiting generalizability of results.

Furthermore, examining sample demographics it becomes apparent that PA of included participants differs from what one could expect of a general student sample. The study conducted by Decamps and colleagues [44] reveals a u-shaped distribution of activity levels: there are many inactive students as well as many active students (>8 h of sports per week). Additionally, Kayani and colleagues [43] report a mean of 3.1 metabolic equivalent (MET)-hours per week, which would be far below average, therefore representing a very inactive student sample.

Regarding sampling time points, Kayani and colleagues [43] and Rettinger and colleagues [42] reported examination of students to take place between October and December, which seems to be at the beginning of the semester. Decamps and colleagues [44] did not report on sampling time. Only Ruthig and colleagues [41] chose to measure pre- and within-examination-stress conditions. The stress level of the sample of Rettinger and colleagues does not differ significantly from a norm sample [43,44,45,46,47,48,49], whereas the samples of Ruthig et al. [50] and Kayani et al. [49] tend to be more stressed, as there is no comparative data available, no assumption can be drawn with regard to the stress level of the sample of Decamps and colleagues.

### 3.6. Results of Individual Studies and Synthesis of Results

While all studies included all three dimensions (PA, AP, and stress), the only relationship reported in all studies was the relation between PA and AP. Three studies analyzed the relationship between stress and AP [41,42,43] and three studies provided results for the relation of PA and stress [42,43,44]. Only one study [43] investigated the relationship of all three variables within a mediation approach. Due to the fact that only one study examined this relationship between all three variables, this relationship was not accounted for in the following meta-analyses. Hence, bidirectional relationships were analyzed and the results merged in narrative synthesis. As only one study used objective measures of PA, and subjective and objective measures are known to produce divergent results (e.g., [51]), only self-reported PA measures were included in analyses. Moreover, self-reported stress as well as objectively documented AP (i.e., GPA) results were included to increase the comparability between the studies, since those were reported in all studies.

Significant results for the relation between PA and AP were reported in one out of the four studies [42] between walking and GPA and between total PA and GPA in a second study [43].

The examination of relationships between PA and stress showed significant results for obligatory exercise and the Inventory of College Students’ Recent Life Experiences (ICSRLE). However, the other measurement tools for Stress (Perceived Stress Scale (PSS)) and PA (International Physical Activity Questionnaire (IPAQ)) did not indicate any statistical significant relation [42]. Both academic and general stress measured by the Freshman-Stress Scale were significantly associated with PA in one study (in the case of general stress, this significant relationship was not present after the transformation to Fisher r-to-z transformed correlation coefficients) [44] as well as general stress measured by the university-stress scale in another [43].

The relationship of AP and stress showed significant results between general stress measured by the ICSRLE and GPA in one study [42] and between general stress measured by the university-stress scale and GPA by another [43]. The effect sizes and more detailed results of the four included studies can be found in Table 1.

### 3.7. Overall Effect Sizes

A meta-analysis of effect sizes was conducted for the relationship between PA and AP, PA and stress, and stress and AP.

**Figure 3:**
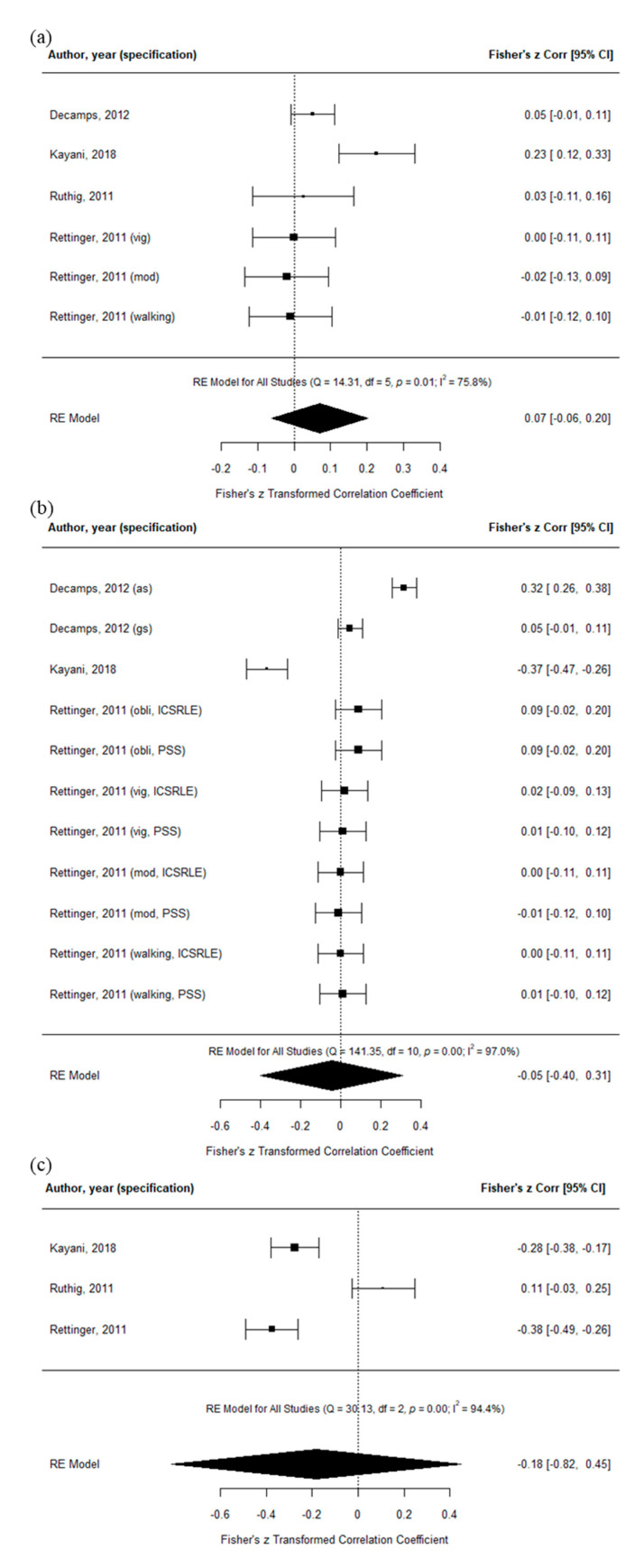
Forest plots of bidirectional relationships. (**a**) Physical activity and academic performance; (**b**) physical activity and stress; (**c**) stress and academic performance. Abbreviations: vig: vigorous physical activity; mod: moderate physical activity; as: academic stress; gs: general stress; obli: obligatory exercise; ICSRLE: Inventory of College Students’ Recent Life Experiences; PSS: perceived stress scale.

The estimated average Fisher r-to-z transformed correlation coefficient based on the random-effects model for the relationship between PA and AP, PA and stress, and stress and AP was 0.07 (95% confidence interval (CI): −0.06–0.20, Q5 = 14.31, *p* = 0.01; I^2^ = 75.8%), −0.05 (95% CI: −0.40–0.31, Q10 = 141.35, *p* < 0.01; I^2^ = 97.0%), and −0.18 (95% CI:−0.82–0.45, Q2 = 30.13, *p* < 0.01; I^2^ = 94.4%), respectively. Therefore, none of the average outcomes of these relationships differed significantly from zero and the I^2^ values indicate significant substantial to considerable heterogeneity. The forest plots for these relationships are displayed in Figure 3.

## 4. Discussion

This systematic review and meta-analysis provided an overview of studies that included PA, stress, and AP in university students. Four studies with more than 1900 participants were identified within this review. The aim was to examine the tridirectional relationship between PA, stress, and AP and to expand upon knowledge on bidirectional relationships of PA and stress, stress and AP, as well as PA and AP, while focusing only on studies assessing all three variables. A mediating or moderating effect of PA on the relation between stress and AP was hypothesized based on theoretical assumptions [7,25]. Even though there were significant relations between the three dimensions in the individual studies, the pooled bidirectional comparisons showed no significant relationship between the dimensions. Only one of the included studies investigated the relationship between all variables of interest: PA, AP, and stress [43]. The remaining three studies also assessed all three variables but only focused on bidirectional relationships. Based on the meta-analytical analyses none of these relationships gained significance and ES indicate small effects for the relation of stress and AP (*z’_StressAP_* = −0.18, <30th percentile), but no meaningful effects for the relations of PA and stress and PA and AP (*z’_PAStress_* = −0.05, *z’_PAAP_*= 0.07, both <15th percentile). Results will be shortly summarized below, starting with the bidirectional relationships, leading to a summary on the tridirectional relationship of PA, Stress and AP.

### 4.1. Bidirectional Relationship between Physical Activity and Academic Performance

Regarding the effects of PA on AP, current literature points to a positive influence of PA on AP in students, as more active students show better AP. However, there is only limited evidence for university student populations [20,52,53], while this association is commonly examined in pupils [18,54,55]. The present review revealed one out of four studies to show a significant effect as well as a non-significant small pooled effect between these parameters in study results. Visual inspection of the forest plots indicate that the results of Kayani and colleagues [43] step out of the line by revealing a significant positive relationship. One reason for this study being the only to reveal this positive relation might be that comprehensive measures for both variables of interest were used, whereas other studies used singe-item measures. However, the average PA was on a low level (3.1 MET-hours per week) in the study of Kayani and colleagues. Therefore, it might be that only participants with low PA differ from those with a higher amount concerning AP. Overall, the proposed positive relationship of PA and AP cannot be confirmed by present results. However, even if results showing the positive influence of PA on AP could not have been replicated, this relation might not be denied as it might be mediated by the positive effect of PA on cognitive performance, which has been repeatedly shown for school children [56,57,58].

### 4.2. Bidirectional Relationship between Physical Activity and Stress

PA is also known to be a stress-buffering mechanism [7]. Inactive participants were formerly shown to have higher stress-related disease incidences than their active counterparts [9], which has also been reported for mental health factors [8]. Paralleling the results of Nguyen-Michel and colleagues [10], the current review revealed a negative, however, non-significant and meaningless relationship between PA and stress pooled over included studies in university students. Interestingly, two out of three studies found significant results, which however point to opposite directions, therefore levelling out in the combined analysis. Whereas Kayani and colleagues [43] found a negative relationship between PA and stress in participants with an overall low PA, Decamps and colleagues [44] found a positive one, meaning that highly active students perceived more academic stress. This contradicts the stress-buffering hypothesis [7]. In this case, however, being active might have been a stressor itself, as increasing time demands for learning activities arose during the examination phase. In this sample, 46% of participants reported a PA of more than 8 h a week making it more challenging to find time to exercise. Based on 168 studies, a systematic review revealed that psychological stress generally predicts less PA [59]. Especially in high-stress periods like examination phases, PA decreases and does therefore not execute its potential as a suitable stress-buffer for students [4].

### 4.3. Bidirectional Relationship between Stress and Academic Performance

The pooled effect for stress and AP revealed a negative small, but non-significant relation, meaning that participants with higher stress have a decreases AP in principle as shown by two out of three studies, which is in line with former studies [15,17]. However, the study conducted by Ruthig and colleagues [41] is out of the line and found a positive relationship and therefore higher stress to elicit better AP. Here, the absolute stress amount of the study population needs to be considered. With a perceived stress scale ranging from 9 to 35, their sample scored around 22 on average (SD = 5.72), showing a high stress amount as compared to the population mean [60,61]. One possible explanation for this result might be that participants who prepared more for the AP also perceived a higher amount of stress but at the same time showed better performance due to their throughout preparation and probably their higher arousal as stated, e.g., by the Individual Zones of Optimal Functioning (IZOF) Theory [62]. Therefore, future studies should assess levels of arousal in students to examine student’s optimal stress zone for optimal AP.

### 4.4. Tridirectional Relationship among Physical Activity, Stress, and Academic Performance

Kayani and colleagues [43] were the only to investigate the relationship of all three variables. They used a parallel mediation model, however, measuring the influence of stress and self-esteem as concurrent mediators of the relationship between PA and AP, with both were found to be significant mediators. Hence, this study is, to the best of our knowledge, the only one to show a mediating effect of stress on the relation of PA and AP, revealing that the higher the amount of PA, the lower the stress level and the higher the stress level, the lower AP. However, even though the mediating effect increases the direct effect of PA on PA, the stress-buffering hypothesis postulates a moderating effect of PA on the relationship of stress and AP, which still remains unclear.

Especially in the examination phase students should have good stress-buffering abilities by performing PA, as high amounts of perceived stress are known to diminish cognitive functioning in students [13]. Moreover, cognitive functioning is highly correlated to AP [14]. The inclusion of all three variables PA, stress, and AP in one statistical (moderation) model might be essential in this context since this might reveal indirect effects not captured by bidirectional comparisons. However, based on theoretical deliberations, it cannot be assured that a mediation approach is the correct underlying mechanism. A moderation approach may also be expedient as also suggested by a review on PA and stress reactivity [63], which has to be examined in future studies by comparing model fits of different approaches.

### 4.5. Limitations

A couple of limitations have to be considered in regard to this review.

First, a meta-analytic analysis of only four (quite heterogenic) studies was performed, whose results has to be viewed with caution. However, the Cochrane Consumers and Communication Review Group [64] stated that as few as two studies are sufficient to conduct a meta-analysis. To account for the small sample, results drawn are less generalizable than results drawn from bigger samples.

From a methodological perspective, the sample size was quite divergent across included studies, ranging from 203 [41] to 1071 [44] and added up to a total of 1952 participants (*n*_female_ = 1220, *n*_male_ = 732) throughout all studies. Individual sample sizes do not appear to be extremely small. However, most studies did not provide any justification for sample size estimation, leaving the question of appropriate sample size and power.

Besides restricted sample sizes, the risk-of-bias assessment revealed three out of the four studies to suffer from a high risk of bias [41,42,44] within studies. Closer inspection revealed that this high risk is commonly caused by not reporting on non-responders. Therefore, more information is needed about non-responders and dropouts in future investigations to be able to better evaluate study quality and weighting results. A similar picture emerged when evaluating risk of bias between studies. Here, a small bias can only be assumed for the relation between PA and AP, whereas the other two suffer from high publication bias. However, this finding was accounted for by using random-effect models for meta-analyses as suggested by the Cochrane Consumers and Communication Group [65]. Nevertheless, findings have to be interpreted with caution, as sources of heterogeneity are unclear. Notably, the studies included different designs and various additional outcomes that were not included in the meta-analysis to achieve a better comparison.

Several other methodological differences between studies made results difficult to compare. Included studies used either cross-sectional [43,44] or longitudinal designs [41] or analyzed longitudinal data cross-sectionally [42]. Moreover, studies differed regarding assessment methods in all three variables of interest: PA is not thoroughly assessed in any of the studies. As two studies used a 7-item short form of the IPAQ [45] measuring PA in different facets [42,43], the two remaining studies only used single-item measures to quantify PA. Here, the next methodological concern arises, as these two studies stated to measure PA, though they explicitly asked for sport and exercise activities, excluding PA like active transportation or gardening, for example. Hence, results regarding PA are difficult to compare between studies. Another concern about terminology arose in the study of Kayani and colleagues [43], who stated to measure depression, but used the University Stress Scale [49] and therefore measured stress to operationalize depression. Moreover, all studies used different, however validated measures for stress. Regarding AP, two studies used GPA as course grade average [42,43], whereas one study used a dichotomous outcome [44] and one used only a specific course grade of a single course [41]. As all studies were interested in influences of or on academic stress, sampling points at the start of a semester, i.e., in a period with only low stress demands, is questionable. If stress is a variable of interest, it should be measured towards the end of a semester (i.e., just before the examination period), where stress demands are known to increase as examinations approach. These differences make results difficult to compare and may have influenced results on relationships of variables of interest.

Last, but not least, a major limitation of the current investigation is that we were not successful in identifying and examining more studies elaborating the tridimensional relationship of PA, stress, and AP, even though we explicitly included only studies with all three variables. Unfortunately, only one of them examined the relation of interest. All other studies focused on bidirectional relationships; thus, not enabling us to draw clear conclusions.

## 5. Conclusions and Future Directions

The current investigation did not evoke any significant relationships between the three variables of interest. Moreover, heterogeneity, the small amount of included studies and above-mentioned limitations prohibited to state clear evidence at this point. Therefore, more studies are needed, expanding upon the investigation of bidirectional relationships and build up upon the study of Kayani and colleagues [43], investigating the tridirectional relationship between PA, stress, and AP. Hence, to encounter the above discussed limitations of the current investigation as well as of existing and included examinations, directions for future research will be systematically compiled below.

(1) Adequate and validated measurement tools should be used. Regarding PA measurement, objective measurement should be the means of choice [66] to conduct a comprehensive quantification of PA. If for feasibility reasons or large sample size requirements PA has to be measured by self-report, validated tools like the IPAQ [45] should be used instead of single items to increase study quality. Regarding stress measurement, a more comprehensive assessment method should be used which also includes objective measures like cortisol to determine real stress exposure as compared to perceived stress because this of the higher relevance from a physiological perspective on stress and health (e.g., [67,68]). Regarding AP measurement, future studies should either use objective measures which can display the overall AP or should collect to the AP data which is directly associated to the measured stress period.
(2) All variables should not only be assessed, but their relations should be analyzed in terms of bi- and tridirectional relationships. Possibly, a theoretical foundation should be used to investigate moderating or mediating effects of one or more variables. To enable secondary data analyses, data should be provided by authors upon request, or should be uploaded for common use in agreement with open science practices.
(3) Future studies should at least control for stressful and non-stressful times during the semester and therefore control for real-life-stress situations or rather experimentally manipulate the perceived amount of stress using randomized controlled designs.
(4) In addition, consistent use of terminology should be strived for to encounter misinterpretation of findings regarding PA, sports and exercise influences.
(5) Encouraging students to be more physically active could be achieved by awareness raising campaigns through lecturers and tutors as well as investments in the sports association and sports facilities at the campus. Following the Okanagan Charter for Health Promoting Universities and Colleges [69], this approach can strengthen student health by forming long term health habits [70]. There are plenty of opportunities to implement PA habits in university students for example by offering sports courses during the examination period or by providing mobile health interventions, which are promising new tools in the area of primary prevention [71].

Taken together, there is currently not enough research available to make reliable statements about the interaction of the three constructs regarding university students. Therefore, it is recommended to conduct further research in this area in order to raise the potential of PA as a predictor for AP under consideration of real life stressors.

## Figures and Tables

**Table 1:** Study characteristics of included studies.

Authors (Year)/Country	Aim	Study Design	Sample Size; Age (SD)	Sample Characteristics/Population	PA Measure	Stress Measure	AP Measure	Direct Association Statistics Transformed Effect Sizes (to Comply with r Values)	Sampling Time; Stress Period?	Central Results	Sub-Findings
Décamps et al. (2012)/France	To examine differences between rare, regular (weekly <8) h and intensive (>8 h) exercising college students on AP, stress, and coping strategies.	cross sectional	1071 (690 female); 18.7 years (no SD provided)	First year students, type of studies not provided	Self-report measure: single item regarding frequency of sports practice in h/week (categorization: less than once a week, less than 8 h per week, or 8 h and more per week)	Self-report measure: Freshmen Stress Scale (Boujut and Bruchon-Schweitzer, 2009); a general score of perceived stress and four specific scores: (1) AS (e.g., "examination revisions” or “new working methods”, (2) stress-related to university disorganization (e.g., “monotony of lessons”, “poor organization within the university”, or “lack of attention from teachers”), (3) loneliness (e.g., “feelings of loneliness” or “difficulties to meet new people”), and (4) social perturbation (e.g., “relational difficulties with family and friends”)	Data by the university administration regarding success vs. failure of moving up into the next academic year	PA to AP: chi-square = 2.82; PA to AS: F = 13.88; PA to GS: F = 9.28 PA to AP: *r* = 0.05; PA to AS: *r* = 0.3065; PA to GS: *r* = 0.0469	Not provided Not controlled for perceived stress	Significant differences between three groups on GS (F(2,1070) = 9.28, *p* <0.001) and AS (F(2,1070) = 13.88, *p* <0.001). No differences in terms of success/failure and sports practice (Chi2 = 2.82, *p* = 0.24)	Rare sports practice (rare and not every often): 406 students (26.4% of male); regular practice (every week but less than 8 h): 173 students (33.5% of male); intensive practice (8 h or more per week): 492 students (43.9% of male)
Kayani et al. (2018)/China	To determine the impact of PA on AP with the mediation of self-esteem and depression (operationalized by stress).	cross sectional	358 (165 female); 20.30 ± 1.15 years	Undergraduate students from five different universities, type of studies not provided	Self-report measure: short version of the International Physical Activity Questionnaire (Craig et al., 2003)	Self- report measure: University Stress Scale (Stallman & Hurst, 2016): index of stress experienced by university students and intensity of stress	GPA for the last consecutive semesters	PA to AP: *r* = 0.222; PA to stress: *r* = −0.352 AP to stress: *r* = −0.269	October–December 2017 Not controlled for perceived stress	Significant association between PA and AP (*r* = 0.222, *p* < 0.01) Significant association between PA and stress (*r* = −0.352, *p* < 0.01) Significant association between AP and stress (*r* = −0.269, *p* < 0.01)	Self esteem; mediation, Indirect EffectsThrough all of the mediators, we can see a strong positive effect for self-esteem (a1b1 = 1.053,CI = 0.456–1.732) and a negative effect for depression (a2b2 = 0.160, CI = −1.841–0.425). This shows that both mediators are significantly associated to PA and AP, because bootstrap CI is above zero while controlling for demographic variables, but most of the indirect effect is due to self-esteem as a1b1 is 1.053 while a2b2 is 0.160.
Rettinger (2011)/USA	To determine the relationship between PA, stress, and AP	Cross sectional	320 (225 female); 19.62 ± 1.6 years	Undergraduate students from religious based institution, type of studies not provided	Self-report measure: short version of the International Physical Activity Questionnaire; (Craig et al., 2003)	Self- report measure: ICSRLE (Kohn 1990); subscales: developmental challenge, time pressure, academic alienation, romantic problems, assorted annoyances, general social mistreatment, and friendship problems PSS (S. Cohen, Tamarck, and Mermelstein, 1983)	Grade Point Average during one semester	PA to AP (vig): ESE = 0; PA to AP (mod): ESE = −0.02; PA to AP (walking): ESE = −0.01PA obli to Stress ICSRLE: ESE = 0.09 PA obli to Stress PSS: ESE = 0.09 PA vig to Stress ICSRLE: ESE = 0.02 PA vig to Stress PSS: ESE = 0.01 PA mod to Stress ICSRLE: ESE = 0 PA mod to Stress PSS: ESE = −0.01 PA walking to Stress ICSRLE: ESE = 0 PA walking to Stress PSS: ESE = 0.01 Stress ICSRLE to AP: ESE = −0.36 ESE = *r*	October–December 2010 Not controlled for perceived stress	Significant association between obli and Stress ICSRLE (ESE = 0.09, *p* < 0.05) Significant association between walking and GPA (ESE = −0.01, *p* < 0.05) Significant association between Stress, ICSRLE and GPA (ESE = −0.36, *p* < 0.05)	Grade point average was significantly related to many demographics
Ruthig et al. (2011)/USA	To examine changes in health perceptions and behaviors among undergraduate college students over an academic year and to determine how such changes impact AP	Cohort	203 (140 female); 18.82 ± 1.50 years	Undergraduate psychology students	Self-report measure: single item regarding frequency of more than 30 min/day PA per week; (categorization: 1 (never) to 7 (seven or more times))	Self- report measure: 7 items from the PSS, sum score (Cohen, Tamarck, and Mermelstein, 1983)	Grade Point Average of introductory psychology course	PA to AP: *β* = 0.025; female *β* = 0.05; male *β* = −0.03 Stress to AP: β = 0.11 female *β* = 0.15; male *β* = 0.02; *β = r*	T1 at the start of the academic year, T2 toward the end (not clearly specified) Not controlled for perceived stress	No significant association found	Physical health symptoms, general psychological health, diet, tobacco use, binge drinking and sleep; gender differences; changes in health perceptions and behaviors

Note. PA: Physical Activity. AP: Academic Performance. GS: General Stress. AS: Academic Stress. Vig: vigorous. Mod: moderate. Obli: obligatory exercise. ICSRLE: Inventory of College Students’ Recent Life Experiences. PSS: Perceived Stress Scale. ESE: Effect size estimate. GPA: Grade Point Average.

**Table 2:** Risk of bias assessment using the Appraisal Tool for Cross-Sectional Studies (AXIS) tool for included studies.

	**AXIS Items 1–10**										
	1_intro_aims	2_methods_study_design	3_ methods_sample_size	4_ methods _defined_population	5_ methods_representation	6_ methods _selection_process	7_methods_non_responders	8_methods_appropriate_measures_aims	9_methods_appropriate_measures_methodogical	10_methods_statistical_indices	
Décamps et al. (2012)/France	Yes	Yes	No	Yes	Yes	No	No	Yes	Yes	Yes	
	See “Introduction”	See “Participants and procedure”	See “Participants and procedure”	First year college students	French university; 381 males and 690 females	See “Participants and procedure”	No information on non-responders	See “Measures” in the methods section	See “Measures” in the methods section	*p* set to 0.05	
Kayani et al. (2018)/China	Yes	Yes	No	Yes	Yes	Yes	Don’t know	Yes	Yes	Yes	
	See “Theoretical background”	See “Measures”	A sample of 358 students was studied.	University students	See “Participants”	See “Participants”	See “Participants”	see “Measures”; Depression = Stress	See “Measures”	*p* set to 0.05; see confidence intervals	
Rettinger (2011)/USA	Yes	Yes	No	Yes	Yes	Yes	No	Yes	Yes	Yes	
	See “Introduction” Part 3	See “Methods”	Three-hundred twenty students responded.	Undergraduate students	See “Participants and recruitment”	An email was sent to the undergraduate student body	No information on non-responders	See “Measures”	See “Survey Instruments”	*p* set to 0.05	
Ruthig et al. (2011)/USA	Yes	Yes	No	Yes	Yes	Yes	No	Yes	Yes	Yes	
	See “The current study”	See “Participants and Procedure“	Participants were 203 undergraduate students	Male and female undergraduate college students	See “Participants and procedure”	see “Participants and procedure”	No information on non-responders	See “Measures”	The Stress: Time 1 Inter-item reliability (α = 0.86)	*p* set to 0.05	
	**AXIS Items 11-20**										
	11_methods_description_overall	12_results_description	13_results_non_response_bias	14_results_information_non_responders	15_results_consistency	16_results_all_analysis	17_discussion_justified_discussion	18_discussion_limitations	19_other_conflict_interest	20_other_ethical_approval	**Score**
Décamps et al. (2012)/France	Yes	Yes	No	No	Yes	Yes	Yes	Yes	No	Yes	**14 (high risk)**
	See “measures” and “analysis of data”	See “Results”	No information on non-responders	No information on non-responders	See “Results”	ANOVA, Tukey post hoc, chi-square	See “Discussion”	See “Limitations”	See “conflict of interest statement”	See “Participants and procedure”	
Kayani et al. (2018)/China	Yes	Yes	No	Yes	Yes	Yes	Yes	Yes	No	Do not know	**15 (moderate risk)**
	See “measures” and “analysis of data”	See “Table 1”	See “Participants”	See “Participants”	See “Results”	EFA, CFA, mediated regression analysis	See “Discussion”	See “Limitations”	The authors declare no conflict of interest.	No information	
Rettinger (2011)/USA	Yes	Yes	No	No	Yes	Yes	Yes	Yes	Don’t know	Do not know	**14 (high risk)**
	See Data Analysis	See “Results”	No information on non-responders	No information on non-responders; 23 did not complete	See “Results”	A univariate general linear model (GLM)	See “Discussion”	See “Limitations”	No information	No information	
Ruthig et al. (2011)/USA	Yes	Yes	No	No	Yes	Yes	Yes	Yes	Don’t know	Do not know	**14 (high risk)**
	see “Methods & Results”	see “Results and Participants”	no information on non-responders	no information on non-responders	see “Results”	ANOVA, *t*-tests, regression analyses	see “Discussion”	see “Limitations”	no information	no information	

## Data Availability

The data that support the findings of this study are available from the corresponding author, upon reasonable request.

## Supplementary Materials

The following are available online at https://www.mdpi.com/1660-4601/18/2/739/s1, Table S1: Individual search terms for databases.
