# Supplementary material for: The Tridirectional Relationship among Physical Activity, Stress, and Academic Performance in University Students: A Systematic Review and Meta-Analysis
Source: Int J Environ Res Public Health. 2021 Jan 16;18(2):739. doi: 10.3390/ijerph18020739 (PMC7830011; doi:10.3390/ijerph18020739)
Supplement: Supplementary file 1 [file ijerph-18-00739-s001.pdf]

Supplement 1.

Supplementary Table 1. Individual search terms for databases.

Database	Searchterm	Matches	Added to project (without dublettes)
**Pubmed**	((((("physical activity"[Title/Abstract] OR "habitual activity"[Title/Abstract] OR "exercise"[Title/Abstract] OR "exercises"[Title/Abstract] OR fitness[Title/Abstract] OR sport[Title/Abstract] OR sedentary[Title/Abstract] OR training[Title/Abstract] OR "physical education"[Title/Abstract] OR "physical activities"[Title/Abstract] OR "habitual activities"[Title/Abstract] OR sports[Title/Abstract] OR sedentariness[Title/Abstract])) AND (stress[Title] OR exam[Title] OR exams[Title] OR test[Title] OR tests[Title] OR "academic performance"[Title])) AND (achievement[Title/Abstract] OR grade[Title/Abstract] OR grades[Title/Abstract] OR performance[Title/Abstract] OR outcome[Title/Abstract] OR attainment[Title/Abstract] OR assessment[Title/Abstract] OR score[Title/Abstract])) AND (student[Title] OR college[Title] OR graduate[Title] OR university[Title] OR tertiary[Title] OR students[Title] OR colleges[Title] OR graduates[Title] OR universities[Title] OR academic[Title])) NOT (school[Title] OR pupil[Title] OR pupils[Title])	399	399
**Scopus**	(TITLE-ABS-KEY ("physical activity" OR "habitual activity" OR "exercise" OR "exercises" OR fitness OR sport OR sedentary OR training OR "physical education" OR "physical activities" OR "habitual activities" OR sports OR sedentariness) AND TITLE (stress OR exam OR exams OR test OR tests OR "academic performance") AND TITLE-ABS KEY (achievement OR grade OR grades OR performance OR outcome OR attainment OR assessment OR score) A ND TITLE (student OR college OR graduate OR university OR tertiary OR students OR colleges OR graduates OR un iversities OR academic) AND NOT TITLE (school OR pupil OR pupils))	1057	507
**SMEI**	ab("physical activity" OR "habitual activity" OR "exercise" OR "exercises" OR fitness OR sport OR sedentary OR training OR "physical education" OR "physical activities" OR "habitual activities" OR sports OR sedentariness) AND ti(stress OR exam OR exams OR test OR tests OR "academic performance") AND ab(achievement OR grade OR grades OR performance OR outcome OR attainment OR assessment OR score) AND ti(student OR college OR graduate OR university OR tertiary OR students OR colleges OR graduates OR universities OR academic) NOT ti(school OR pupil OR pupils)	204	81
**ERIC**	("physical activity" OR "habitual activity" OR "exercise" OR "exercises" OR fitness OR sport OR sedentary OR training OR "physical education" OR "physical activities" OR "habitual activities" OR sports OR sedentariness) AND +title:(stress OR exam OR exams OR test OR tests OR "academic performance") AND (achievement OR grade OR grades OR performance OR outcome OR attainment OR assessment OR score) AND +title:(student OR college OR graduate OR university OR tertiary OR students OR colleges OR graduates OR universities OR academic) -school -pupil -pupils	208	185

**Web of Science**	TOPIC: ("physical activity" OR "habitual activity" OR "exercise" OR "exercises" OR fitness OR sport OR sedentary OR training OR "physical education" OR "physical activities" OR "habitual activities" OR sports OR sedentariness) AND TITLE: (stress OR exam OR exams OR test OR tests OR "academic performance") AND TOPIC: (achievement OR grade OR grades OR performance OR outcome OR attainment OR assessment OR score) AND TITLE: (student OR college OR graduate OR university OR tertiary OR students OR colleges OR graduates OR universities OR academic) NOT TITLE: (school OR pupil OR pupils)	721	580
		**Total:**	**1752**
